# Supplementary material for: Current Evidence on Vasa Previa without Velamentous Cord Insertion or Placental Morphological Anomalies (Type III Vasa Previa): Systematic Review and Meta-Analysis
Source: Biomedicines. 2023 Jan 7;11(1):152. doi: 10.3390/biomedicines11010152 (PMC9856204; doi:10.3390/biomedicines11010152)
Supplement: Supplementary file 1 [file biomedicines-11-00152-s001.zip › Supplemental files_R1.pdf]

Supplemental Table S1. Definition of heterogeneity among the eligible studies.

I^2 value	Heterogeneity	Analysis
1-29%	Low	Fixed-effect
30–60%	Moderate	Random-effect
50–90%	Substantial	Random-effect
75–100%	Considerable	Random-effect

According to the *Cochrane Handbook for Systematic Reviews of Interventions* (ver 6.3), heterogeneity was determined per the I^2 value [1].

Supplemental Table S2. Risk of bias assessment for the comparator study.

Authors	Confounding	Selection	Classification of intervention	Deviations from interventions	Missing data	Measurement of outcomes	Reported results	Overall bias
Kamijo K [2]	● Serious	● Serious	● Moderate	● No information	● No information	● Moderate	● Moderate	● Serious
Kanda E [3]	● Serious	● Serious	● Moderate	● No information	● No information	● Moderate	● Serious	● Serious

Risk of bias assessment was performed using the Risk Of Bias In Non-randomized Studies–of Interventions tool (ROBINS-I) [4-6].

● Low risk of bias (the study is comparable to a well-performed randomized trial with regard to this domain)

● Moderate risk of bias (the study is sound for a non-randomized study with regard to this domain but cannot be considered comparable to a well-performed randomized trial)

● Serious risk of bias (the study has some important problems in this domain)

● Critical risk of bias (the study is too problematic in this domain to provide any useful evidence on the effects of intervention.

● No information on how to base a judgment on the risk of bias for this domain.

References

[1] Cochrane Handbook for Systematic Reviews of Interventions. Version 6.1, 2020. Chapter 10: Analysing data and undertaking meta-analyses. https://training.cochrane.org/handbook/current/chapter-10. (accessed 12/20/2022).

[2] Kamijo K, Miyamoto T, Ando H, Tanaka Y, Kikuchi N, Shinagawa M, et al. Clinical characteristics of a novel "Type 3" vasa previa: case series at a single center. The journal of maternal-fetal & neonatal medicine : the official journal of the European Association of Perinatal Medicine, the Federation of Asia and Oceania Perinatal Societies, the International Society of Perinatal Obstet. 2022;35:7730-6.

[3] Kanda E, Matsuda Y, Kamitomo M, Maeda T, Mihara K, Hatae M. Prenatal diagnosis and management of vasa previa: a 6-year review. The journal of obstetrics and gynaecology research. 2011;37:1391-6.

[4] Sterne JA, Hernan MA, Reeves BC, Savovic J, Berkman ND, Viswanathan M, et al. ROBINS-I: a tool for assessing risk of bias in non-randomised studies of interventions. BMJ. 2016;355:i4919.

[5] Danna SM, Graham E, Burns RJ, Deschenes SS, Schmitz N. Association between Depressive Symptoms and Cognitive Function in Persons with Diabetes Mellitus: A Systematic Review. PLoS One. 2016;11:e0160809.

[6] ROBINS-Idetailed guidance (2016). https://www.riskofbias.info/welcome/home/current-version-of-robins-i/robins-i-detailed-guidance-2016. (accessed 12/20/2022).
